# Effect of Interfacial Structure on Mechanical Properties of Graphene Reinforced Al_2_O_3_–WC Matrix Ceramic Composite

**DOI:** 10.3390/nano11061374

**Published:** 2021-05-22

**Authors:** Xuchao Wang, Jun Zhao, Enzhao Cui, Xianhua Tian, Zhefei Sun

**Affiliations:** 1 Key Laboratory of High Efficiency and Clean Mechanical Manufacture of MOE, School of Mechanical Engineering, Shandong University, Jinan 250061, China; wangxuchao@mail.sdu.edu.cn (X.W.); 201612688@mail.sdu.edu.cn (E.C.); xhtian2011@163.com (X.T.); sunzf@mail.sdu.edu.cn (Z.S.); 2 National Demonstration Center for Experimental Mechanical Engineering Education, School of Mechanical Engineering, Shandong University, Jinan 250061, China; 3 School of Mechatronic Engineering, China University of Mining and Technology, Xuzhou 221116, China

**Keywords:** ceramic composite, graphene, interface, first-principles, mechanical properties

## Abstract

The interfacial structures and interfacial bonding characteristics between graphene and matrix in graphene-reinforced Al_2_O_3_–WC matrix ceramic composite prepared by two-step hot pressing sintering were systematically investigated. Three interfacial structures including graphene–Al_2_O_3_, graphene–Al_2_OC and graphene-WC were determined in the Al_2_O_3_–WC–TiC–graphene composite by TEM. The interfacial adhesion energy and interfacial shear strength were calculated by first principles, and it has been found that the interfacial adhesion energy and interfacial shear strength of the graphene–Al_2_OC interface (0.287 eV/nm^2^, 59.32 MPa) were far lower than those of graphene–Al_2_O_3_ (0.967 eV/nm^2^, 395.77 MPa) and graphene–WC (0.781 eV/nm^2^, 229.84 MPa) interfaces. Thus, the composite with the strong and weak hybrid interfaces was successfully obtained, which was further confirmed by the microstructural analysis. This interfacial structure could induce strengthening mechanisms such as load transfer, grain refinement, etc., and toughening mechanisms such as crack bridging, graphene pull-out, etc., which effectively improved mechanical properties.

## 1. Introduction

Graphene has been proven to be an ideal reinforcement to fabricate Al_2_O_3_-based ceramic composites because of its large specific surface, high aspect ratio, superior mechanical, thermal and electrical properties [1,2]. Grigoriev et al. [3] used graphene to reinforce Al_2_O_3_–SiCw ceramics, and they found that the composite with 0.5 vol% graphene showed the optimal flexural strength (904 ± 56 MPa), fracture toughness (10.6 ± 0.3 MPa·m^1/2^) and hardness (22 ± 0.8 GPa), respectively increased by 29%, 10% and 4% compared to the Al_2_O_3_–SiCw composite. Cheng et al. [4] used graphene platelets (GPLs) to reinforce Al_2_O_3_/TiC ceramic composites, and they found that the optimal fracture toughness (8.7 ± 0.4 MPa·m^1/2^) was obtained when the content of GPLs was 0.2 wt%, which increased by 67.3% compared to the Al_2_O_3_/TiC ceramic. Wang et al. [5] fabricated Al_2_O_3_/WC/TiC/graphene ceramic composites, and they found that the optimal flexural strength and fracture toughness were 646.31 ± 20.78 MPa, 9.42 ± 0.40 MPa·m^1/2^, respectively, when the content of graphene was 0.5 vol%, which were significantly improved compared to the composite without graphene.

Interfaces of graphene-reinforced composites are of great significance to their performances [6]. The strong bonding interface is beneficial to improve load transfer efficiency, thus improving strength of composites [7]. Mu et al. [8] investigated the relationship between interfacial strength and strengthening efficiency based on the shear-lag model, and they found that the high interfacial strength improved strengthening efficiency and enhanced the strength of multilayer graphene (MLG)/Ti composites. However, the weak bonding interface can promote graphene pull-out from the matrix, consequently leading to a significant improvement in fracture toughness [9]. Cheng et al. [10] introduced weak interfaces into ZrB_2_–SiC matrix composite ceramics by incorporating graphene, and they found that the interfaces could induce multiple toughening mechanisms including crack deflection, microcracks, graphene bridging and pull-out. Bódis et al. [9] found that nanopores developed at the Si_3_N_4_–MLG interfaces improved the fracture toughness of Si_3_N_4_–MLG composites; however, excessive nanopores led to a lower fracture toughness. Composites with strong and weak bonding interfaces simultaneously can induce microcracks at the interfaces, thus improving strength and toughness [11,12]. Sun et al. [13] found that excellent mechanical properties were achieved in MLG reinforced binderless TiC nanocomposite cemented carbide, and multi-phase and multi-scale strong–weak hybrid interfaces between MLG and matrix grains were responsible for the excellent mechanical properties.

The formation of strong and weak hybrid interfaces mainly depends on regulating interfacial bonding strength. And formation of interfacial phases induced by interfacial chemistry reactions is an effective method to regulate interfacial bonding strength. Ju et al. [7] found that the formation of Al_2_O_3_ interfacial phase at the interface between Al and graphene effectively improved the interfacial bonding strength of the Al–graphene interface, which further improved mechanical properties of composites. Chu et al. [6] found that CuCr could react with reduced graphene oxide (RGO) to form Cr_7_C_3_ at the CuCr–RGO interface, which improved interfacial bonding strength. However, in the field of graphene-reinforced Al_2_O_3_–WC matrix ceramics, interfacial phases and interfacial bonding characteristics between graphene and matrix grains have not been explored. Furthermore, the relationship among interfacial structure, mechanical properties and strengthening and toughening mechanisms in Al_2_O_3_–WC matrix ceramic composites also needs to be further understood.

In order to more thoroughly investigate interfacial bonding characteristics, some theories such as density functional theory (DFT) were more widely used in the interfacial investigation of composites. Zhang et al. [14] studied the interfacial interaction between graphene and copper with a different interfacial oxygen level using first-principles total energy calculations based on DFT, and the results indicated that the increasing oxygen level in the matrix-side interface was less effective in improving the Cu–graphene interfacial bonding, and they verified that the DFT calculation results matched well with the experimental results. Goyenola et al. [15] studied energetic and structural aspects of fullerene-like sulfocarbide (CS_x_) by DFT simulations during synthetic growth with C_m_S_n_ (m, n ≤ 2), and they successfully predicted the range of S concentrations appropriate for well-structured FL–CS_x_. From the above description, it can be concluded that DFT methods can provide significant help to analyze interfacial structures and other features of composites at the atomic level. However, in the field of Al_2_O_3_–WC matrix ceramic composites, the related reports are few.

Two-step sintering is reckoned as an effective method to produce ceramic composites, because this sintering method can suppress ceramic grain growth while fully densifying ceramic composites [16,17]. In the first-step sintering, the green body is heated to a high temperature (T_1_) with short dwell time or without dwell time, which is beneficial to obtain a critical relative density and makes pores unstable [18]. In the second-step sintering, the sintering sample is cooled to a lower temperature (T_2_) with long dwell time; during this stage, grain boundary migration is frozen whereas grain boundary diffusion is active, which is beneficial to inhibiting grain growth while promoting densification [13]. In addition, the long dwell time in the second-step sintering can guarantee the completion of interfacial chemistry reaction and the formation of stable interfacial phases. Lin et al. [19] respectively employed a two-step sintering technique and a traditional sintering technique to fabricate Al_2_O_3_ ceramics, and they found that a measurably higher final density, a smaller average grain size and a narrower distribution in grain sizes were achieved with the two-step sintering technique.

In this work, the Al_2_O_3_–WC–TiC and Al_2_O_3_–WC–TiC–graphene composites were prepared by two-step hot pressing sintering, and the microstructures and mechanical properties of the two composites were evaluated. Especially, the interfacial structures were analyzed by TEM, and the interfacial adhesion energy and interfacial shear strength of Al_2_O_3_–graphene, Al_2_OC–graphene and WC–graphene were calculated by first principles. In addition, the strengthening and toughening effects in ceramic composites induced by the strong and weak hybrid interfaces were discussed.

## 2. Experimental Procedure

### 2.1. Preparation of Composite Powders

Polyvinyl pyrrolidone (PVP, Kemiou Chemical Reagent Co., Ltd., Tianjin, China) was chosen as dispersion medium to disperse graphene. PVP powders were firstly dispersed in the absolute ethyl alcohol for 1 h to get stable graphene dispersion. After that, graphene (thickness: ~0.8 nm, lateral size: 0.5–5 μm, specific surface area: 500–1000 m^2^/g, purity: 99%, XFNANO Materials Technology Co., Ltd., Nanjing, China) was dispersed for 1 h in this dispersion. Polyethylene glycol (PEG, Sinopharm Chemical Reagent Co., Ltd., Shanghai, China) was used as dispersion media to disperse nanosized TiC powders. TiC (40 nm, 99.99% TiC) powders were first dispersed in the absolute ethyl alcohol for 10 min, and then PEG was added into the TiC slurry, and the slurry was further dispersed for 20 min. The dispersed graphene slurry and TiC slurry were mixed, and Al_2_O_3_ powders (0.5 μm, 99.99% Al_2_O_3_), WC powders (0.4 μm, 99.7% WC), MgO powders (0.4 μm, 99.9% MgO), Y_2_O_3_ powders (0.5 μm, 99.9% Y_2_O_3_) and Co powders (0.6 μm, 99.9% Co) were added into the mixed slurry. The mixed slurry was dispersed for 1 h to get stable hybrid slurry. It is important to point out that the Al_2_O_3_, WC, TiC, MgO, Y_2_O_3_ and Co powders were all provided by Chaowei Nanotechnology Co., Ltd., Shanghai, China. It is also important to point out that MgO and Y_2_O_3_ were used as sintering aids and Co was used as metal binder. After dispersion, wetting ball milling and vacuum drying were conducted on the hybrid slurry to get composite powders. Then, the composite powders were screened using a 200-mesh sieve.

### 2.2. Two-Step Hot Pressing Sintering

The prepared composite powders were sintered using two-step sintering method in a hot pressing (HP) furnace (ZRC85-25T, Shanda Nonferrous Metal Casting Co., Ltd., Jinan, China). Firstly, the composite powders were heated to 1750 °C at a heating rate of 40 °C/min without dwell, and then they were rapidly cooled to 1600 °C for 15 min dwell time; finally, they were cooled to the room temperature. It is important to point out that the 1600 °C dwell temperature is determined according to our previous report in literature [20], because the higher mechanical properties of Al_2_O_3_–WC matrix composite can be obtained in this dwell temperature. The sintering pressure was maintained at 32 MPa during the dwell process. In our previous report [5], we proved that the mechanical properties of graphene reinforced Al_2_O_3_–WC–TiC ceramic composites were optimal when the content of graphene was 0.5 vol%. Therefore, in this work, only two ceramic composites, named AWTG0.5 and AWTG0, were prepared and their compositions were shown in Table 1.

### 2.3. Characterization

After cutting, grinding and polishing, dimensions of the test specimens were 3 mm × 4 mm × 30 mm. The bulk density of specimens was obtained by Archimedes method and the theoretical density was calculated by the mixing rule. The average grain size of specimens was obtained by the linear intercept method, and at least 5 SEM images and 300 ceramic grains were included for each specimen. Flexural strength was measured by three-point bending method operated on an electromechanical universal testing machine (CMT6203, MTS Systems (China) Co., Ltd., Shenzhen, China), and the span and loading velocity were 20 mm and 0.5 mm/min, respectively. At least five specimens were tested for each composite during the three-point bending experiment. Vickers hardness was obtained by a Vickers hardness tester (MHVD-50AP, Jüjing Precision Instrument Manufacturing Co., Ltd., Shanghai, China). The load and dwell time were 20 kg and 15 s, respectively. Fracture toughness of specimens was obtained by the indentation method, its value was calculated by the following equation [21]:

(1) $$K_{\mathrm{IC}}=0.016\cdot (\frac{E}{H})\cdot \frac{P}{c^{3/2}}$$

where *E* is the Young’s modulus of composites, *H* is the indentation hardness, *P* is the indentation load, and *c* is the half length of indentation crack. At least 10 indentations were performed on each composite during the indentation experiment.

Phase compositions of specimens were analyzed by the Rigaku Ultima IV X-ray diffraction (XRD, Rigaku, Tokyo, Japan) device. The graphene structures were examined by a HORIBA LabRAM HR Evolution Raman spectroscopy (HORIBA Scientific, Paris, France). Microstructures of specimens were observed by a JEOL JSM-7610F field emission scanning electron microscopy (FE-SEM, JEOL, Tokyo, Japan) and a ZEISS Gemini 300 FE-SEM (ZEISS, Oberkochen, Germany). Interfacial structures were analyzed by a FEI Talos F200 transmission electron microscopy (TEM, FEI, Hillsboro, USA) and a Talos F200X G2 TEM (Thermo Fisher Scientific, Waltham, USA).

## 3. Results and Discussion

### 3.1. Microstructures

**Figure 1:**
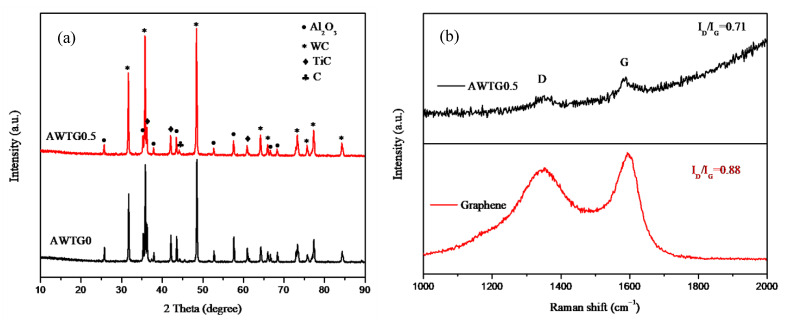
(**a**) XRD patterns of AWTG0 and AWTG0.5; (**b**) Raman spectra of graphene in AWTG0.5 and starting graphene.

Figure 1a shows XRD patterns of AWTG0 and AWTG0.5. The phases of Al_2_O_3_, WC and TiC are detected in the two composites, however, graphene is not detected in AWTG0.5, which may be related to the low volume fraction of graphene. Figure 1b shows Raman spectra of graphene in AWTG0.5 and starting graphene. D and G peaks can be detected in the Raman spectrum of AWTG0.5, which indicates that the graphene structure is reserved in the ceramic composite after two-step HP. Graphene structure stability is generally evaluated by the intensity ratio of D peak to G peak (I_D_/I_G_), and a higher I_D_/I_G_ value denotes the higher structure defect [22,23]. As shown in Figure 1b, the I_D_/I_G_ value of AWTG0.5 is slightly lower than that of staring graphene, which indicates that a slight agglomeration occurs during the dispersing or ball milling process, decreasing the edge defects of graphene [24].

**Figure 2:**
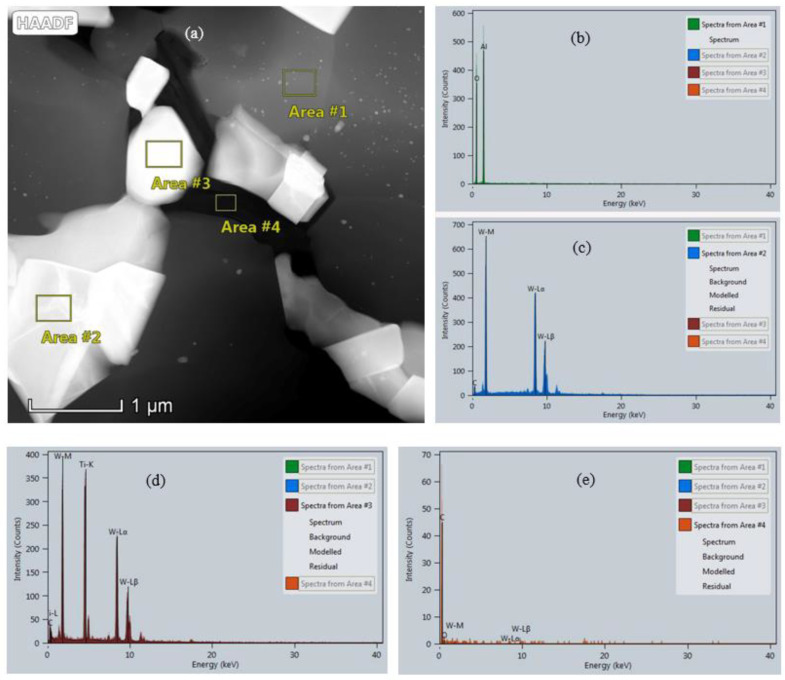
(**a**) HADDF-STEM image of AWTG0.5. EDS spectra of (**b**) Area #1, (**c**) Area #2, (**d**) Area #3 and (**e**) Area #4 labeled in (**a**).

Figure 2 shows the HADDF-STEM image of AWTG0.5 and EDS spectra of the labelled areas. It can be concluded that Area #1, Area #2, Area #3 and Area #4 correspond to the phases of Al_2_O_3_, WC, (W, Ti)C and graphene, respectively. The (W, Ti)C solid solution may result from a part of W atoms dissolving into TiC lattices during sintering process. Meanwhile, the free carbon is left, which can be found in XRD patterns shown in Figure 1a. Additionally, the (W, Ti)C solid solution exhibits the crystal diffraction characteristics of TiC during XRD examination. Therefore, it is not detected in XRD examination.

**Figure 3:**
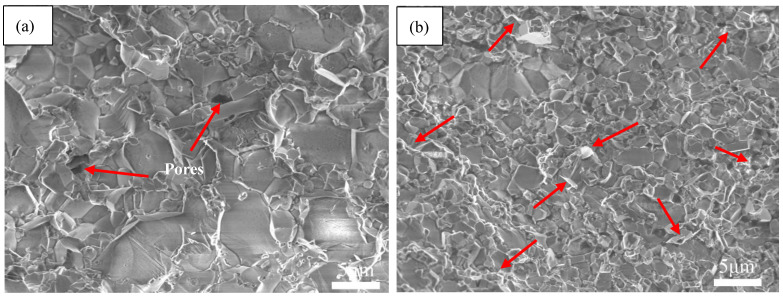
SEM morphologies of fractured surfaces of (**a**) AWTG0 and (**b**) AWTG0.5. Arrows labelled in (**a**) indicate pores and arrows labelled in (**b**) indicate graphenes.

Figure 3 shows SEM morphologies of fractured surfaces of AWTG0 and AWTG0.5. As shown in Figure 3a, ceramic grains of AWTG0 are relatively coarse and some large pores can be clearly observed. As shown in Figure 3b, graphene is homogeneously distributed in the AWTG0.5 composite, meanwhile, ceramic grains of AWTG0.5 are refined and no large pores can be observed in the microstructure of AWTG0.5. The relative densities and average grain sizes of AWTG0 and AWTG0.5 are shown in Table 2. It can be concluded that the ceramic composite incorporating with graphene can effectively improve densification and suppress grain growth. The improved relative density can be attributed to the high thermal and electrical conductivity of graphene, which can improve the heat distribution and further improve sintering performance during two-step HP [13,25]. The refined ceramic grains can be attributed to the pinning effect of graphene on ceramic grains and strong bonding interfaces between graphene and ceramic grains [26].

**Figure 4:**
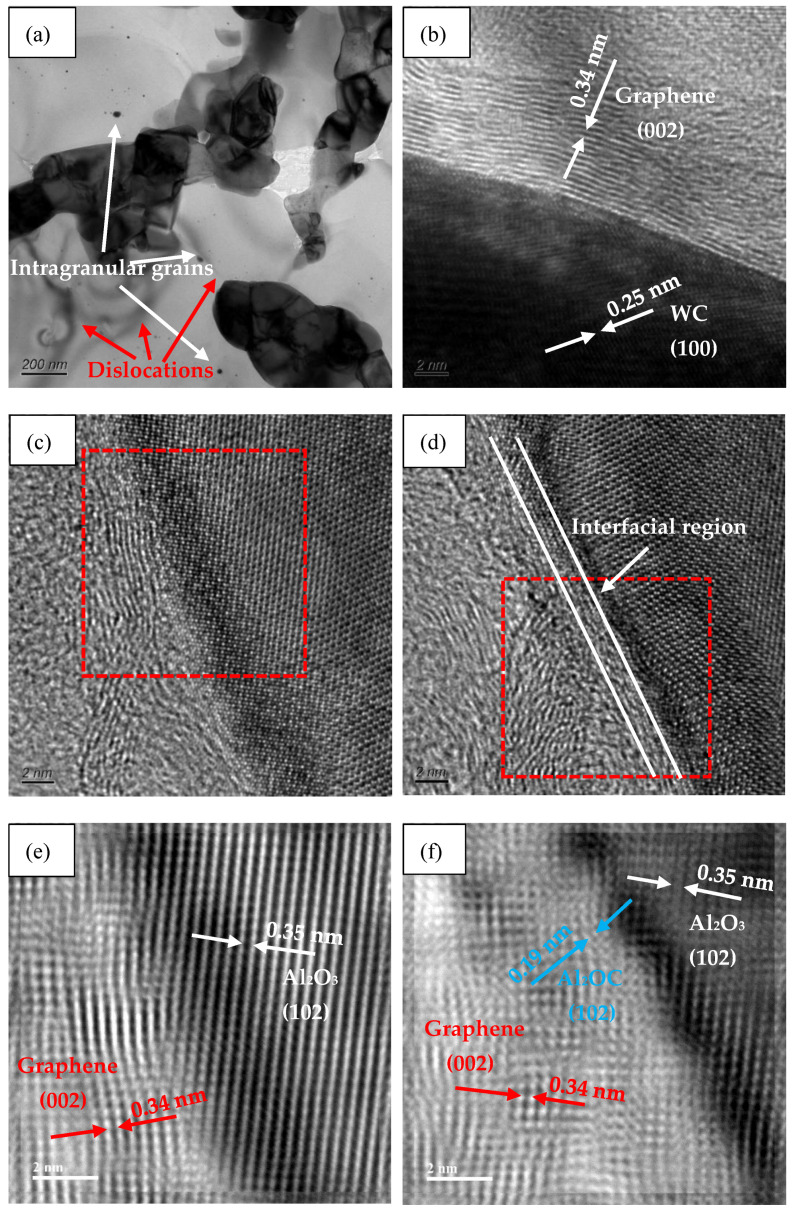
Interfacial structures between graphene and matrix in AWTG0.5. (**a**) TEM image. (**b**) HRTEM image of graphene–WC interface. (**c**,**d**) HRTEM images of graphene–Al_2_O_3_ interface. (**e**,**f**) IFFT images of box regions labelled in (**c**,**d**).

Figure 4 shows the interfacial structures between graphene and matrix in AWTG0.5. As shown in Figure 4a, graphene is distributed at matrix grain boundaries and it is well bonded with matrix grains. Meanwhile, some intragranular (W, Ti)C solid solution grains are distributed in Al_2_O_3_ matrix. In addition, some dislocations near intragranular grains can be clearly observed. It has been reported that the intragranular structure and accumulation of dislocations are beneficial to improving the mechanical properties of ceramic composites [27,28,29].

The interfacial structure between WC and graphene is shown in Figure 4b. It can be seen that graphene is well bonded with WC and the interface is free from grooves, pores or gaps. The WC phase and graphene can respectively be identified by the lattice spacing of 0.25 nm corresponding to the (100) plane and the lattice spacing of 0.34 nm corresponding to the (002) plane. The interface between WC and graphene exhibits a directly physical bonding without interfacial chemical compounds, which is beneficial to enhancing the load transfer efficiency and further improving the strength of ceramic composites [14].

The interfacial structures between Al_2_O_3_ and graphene are shown in Figure 4c,d. It can be seen that graphene is also well bonded with Al_2_O_3_ and the interfaces are free from grooves, pores and gaps. Furthermore, the interfaces between Al_2_O_3_ and graphene show different interfacial characteristics. The interface shown in Figure 4c is a physical bonding interface without interfacial chemistry compounds. Figure 4e shows the inverse fast Fourier transform (IFFT) image of the box region labeled in Figure 4c, which can provide strong evidence that the interface shown in Figure 4c is composed of Al_2_O_3_ and graphene without interfacial chemical compounds. However, as shown in Figure 4d, an approximately 2 nm thick interfacial transition region is found between graphene and Al_2_O_3_. Figure 4f shows the IFFT image of the box region labelled in Figure 4d; it can be determined that the lattice spacing of the interfacial region is 0.19 nm, which is in accordance with the (102) plane of Al_2_OC. The formation of Al_2_OC can be attributed to the following chemical reactions [30,31]:

$${\mathrm{Al}}_{2}O_{3}+2C\Leftrightarrow {\mathrm{Al}}_{2}O_{\left(g\right)}+2{\mathrm{CO}}_{\left(g\right)}$$

$${\mathrm{Al}}_{2}O_{\left(g\right)}+C\Leftrightarrow {\mathrm{Al}}_{2}\mathrm{OC}$$

Al_2_O_3_ grains in the presence of graphene undergo a carbothermal reduction to form gaseous Al_2_O which further reacts with residual carbon to form Al_2_OC.

In our previous work [32], we prepared Al_2_O_3_-WC-TiC-graphene ceramic composite using traditional HP sintering technology at 1700 °C for 10 min dwell time, and we found that interfacial phases of Al_4_O_4_C and Al_4_C_3_ were formed between graphene and Al_2_O_3_. Comparing the present work and the previous work in reference [32], it can be confirmed that the different preparation technology could provide different interfacial chemistry reaction products. In fact, the different interfacial phases in the two works can be mainly attributed to the different dwell temperatures in the two preparation technologies. It has been reported [31] that a low dwell temperature (1500~1600 °C) was beneficial to producing Al_2_OC at the interface between graphene and Al_2_O_3_, however, a higher dwell temperature was beneficial to producing Al_4_O_4_C and Al_4_C_3_, which is in agreement with our research results.

**Figure 5:**
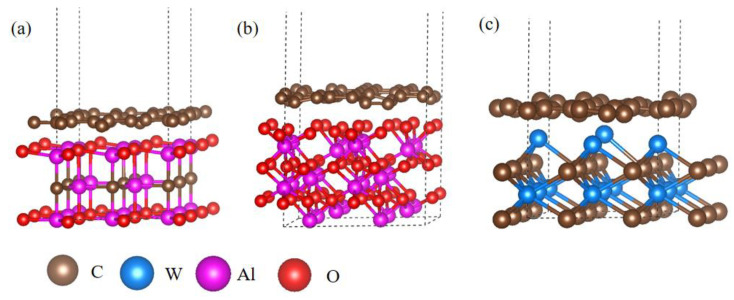
Interface models of (**a**) Al_2_OC–graphene, (**b**) Al_2_O_3_–graphene and (**c**) WC–graphene.

The interfacial bonding characteristics between graphene and matrix grains were further analyzed by first-principles calculations based on density functional theory (DFT). The calculations were performed by VASP and the detailed calculation method is depicted in our previous report [32]. It is important to point out that the first-principles calculations and related results correspond to the ground state instead of accounting for finite temperatures. The as-built interfacial structures including graphene–Al_2_OC, graphene–Al_2_O_3_ and graphene–WC interfaces are shown in Figure 5. The supercell dimensions are 7.17 × 7.17 × 20.07 Å^3^, 9.73 × 9.73 × 20.93 Å^3^ and 6.62 × 6.62 × 19.46 Å^3^, respectively. After geometry optimization, the equilibrium distances between the graphene and the top of the Al_2_OC surface, the graphene and the top of the Al_2_O_3_ surface and the graphene and the top of the WC surface are 2.08 Å, 1.78 Å and 1.84 Å, respectively. A 15 Å vacuum layer is used in every calculation model to prevent interactions between the periodic layers [33].

The adhesion energy was calculated by the following equation [7]:

(2) $$W_{\mathrm{ad}}=(E_{\mathrm{graphene}/\mathrm{matrix}}-E_{\mathrm{graphene}}-E_{\mathrm{matrix}})/A$$

where *E*_graphen/matrix_ is the total energy of interfacial structure, *E*_graphene_ and *E*_matrix_ are the surface energy of graphene and matrix, *A* is the area of the interface. In order to calculate the interfacial shear strength, the conjugate gradient optimization method was used to relax the atomic positions by keeping the cell size fixed. With the increase of shear displacement, the shear stress increased until it reached a maximum value, and the shear strength was calculated at the maximum shear value. The calculated results of the adhesion energy and interfacial shear strength of graphene–Al_2_OC, graphene–WC and graphene–Al_2_O_3_ interfaces are shown in Table 3. It should be pointed out that the calculated results of graphene–Al_2_O_3_ and graphene–WC were reported in our previous work [32]. It can be seen that the adhesion energy (0.287 eV/nm^2^) and interfacial shear strength (59.32 MPa) of the graphene–Al_2_OC interface are much lower than those of graphene–WC and graphene–Al_2_O_3_ interfaces, which indicates that the weak chemical bonding is generated at the graphene–Al_2_OC interface and the load transfer efficiency will be affected. However, the high adhesion energy of graphene–WC (0.781 eV/nm^2^) and graphene–Al_2_O_3_ (0.967 eV/nm^2^) interfaces indicates the strong bonding interfaces are generated in the composite. Furthermore, the high interfacial shear strength of graphene–WC (229.84 MPa) and graphene–Al_2_O_3_ (395.77 MPa) interfaces is beneficial to improving load transfer efficiency, further improving mechanical properties of AWTG0.5 [34]. The formation of graphene–Al_2_OC, graphene–WC and graphene–Al_2_O_3_ interfaces indicates that the composite with the strong and weak hybrid interfaces is successfully obtained.

**Figure 6:**
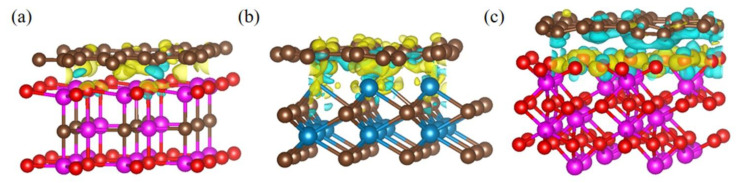
3D charge density difference for (**a**) graphene–Al_2_OC interface, (**b**) graphene–WC interface and (**c**) graphene–Al_2_O_3_ interface. Yellow regions represent the charge accumulation and blue regions represent the charge depletion.

The charge density difference (Δ*ρ*) can be used to analyze interfacial bonding characteristics by charge interaction, and it can be calculated by the following equation [35]:

(3) $$\Delta \rho =\rho_{\mathrm{AB}}-\rho_{A}-\rho_{B}$$

where *ρ*_AB_, *ρ*_A_ and *ρ*_B_ denote the charge density of AB hybrid system, isolated A system and isolated B system, respectively. After geometry optimization and calculation, the 3D charge density differences for graphene–Al_2_OC, graphene–WC and graphene–Al_2_O_3_ interfaces with the isovalue of 0.003 e/Å^3^ are shown in Figure 6. Yellow and blue regions represent charge accumulation and charge depletion, respectively. As shown in Figure 6a, the charge accumulation mainly concentrates at the interface, which indicates that the graphene–Al_2_OC interface is characterized by the covalent bonding [36]. As shown in Figure 6b, the charge accumulation mainly concentrates at the interface and the graphene surface, which indicates that the graphene–WC interface is characterized by the mixture of covalent bonding and ionic bonding [37]. As shown in Figure 6c, the charge accumulation and charge depletion are mainly concentrated at the Al_2_O_3_ surface, therefore, we can guess that graphene may change electron distribution of Al_2_O_3_ surface when the graphene and Al_2_O_3_ interfaces are in contact, which promotes the interaction between graphene and Al_2_O_3_. In addition, as shown in Figure 6a–c, the charge accumulation or depletion density of the graphene–WC and graphene–Al_2_O_3_ interfaces is obviously higher than that of graphene-Al_2_OC, which indicates that the charge interaction of the graphene–WC and graphene–Al_2_O_3_ interfaces is stronger than that of the graphene–Al_2_OC interface, and further indicating that the two interfaces possess the higher interfacial bonding strength [38].

**Figure 7:**
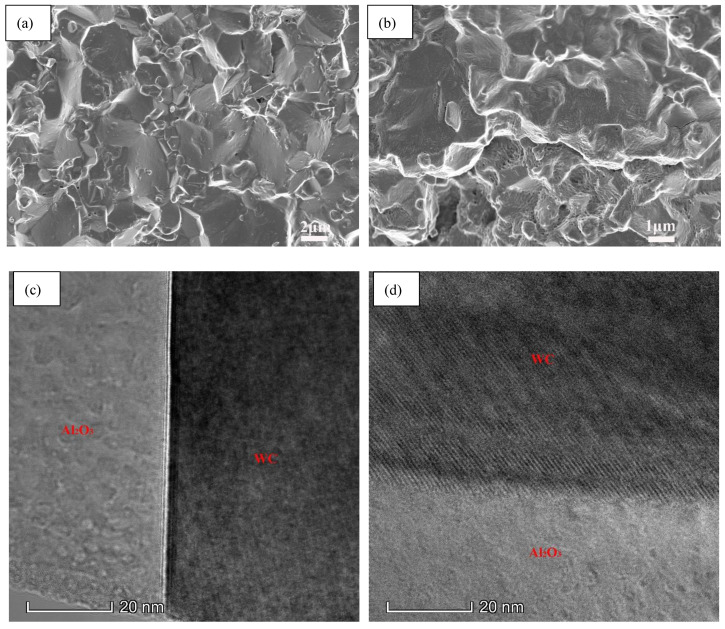
High magnification SEM images of fractured surfaces of (**a**) AWTG0 and (**b**) AWTG0.5. Interfacial structures between Al_2_O_3_ and WC in (**c**) AWTG0 and (**d**) AWTG0.5.

Figure 7a,b show high magnification SEM images of fractured surfaces of AWTG0 and AWTG0.5. As shown in Figure 7a, the fractured mode of AWTG0 is intergranular fracture, however, as shown in Figure 7b, the fractured mode of AWTG0.5 is a mixed fracture mode of intergranular fracture and transgranular fracture. Generally, in ceramic composites, cracks are prone to propagate along the grain boundaries if the boundaries are weaker than the grains [39]. The intergranular fracture mode of AWTG0 indicates that the interfaces between different grains are characterized by weak bonding in AWTG0. Figure 7c shows the interfacial structure between Al_2_O_3_ and WC in AWTG0; it can be seen that a clear groove is distributed along the interface and the groove can greatly weaken the interfacial bonding strength, which further demonstrates that the interfaces in AWTG0 are characterized by weak bonding. The mixed fracture mode of intergranular fracture and transgranular fracture in AWTG0.5 indicates that the strong and weak hybrid interfaces are formed in the composite, which is consistent with the conclusion drawn by the first-principles calculations. Figure 7d shows the interfacial structure between Al_2_O_3_ and WC in AWTG0.5; it can be seen that the Al_2_O_3_ grain and WC grain are physically and tightly bonded without interfacial phases. The different interfacial bonding characteristics between Al_2_O_3_ and WC in AWTG0 and AWTG0.5 can be attributed to the introduction of graphene in ATWG0.5, which improves the grain boundary diffusion by improving the distribution of current and heat in the composite [13]. In addition, the physical and tight bonding interface is beneficial to enhancing load transfer efficiency and improving interfacial bonding strength [14,40]. Therefore, it can be further confirmed that the weak bonding interfaces in AWTG0.5 are induced by interfacial chemical reaction between graphene and Al_2_O_3_.

### 3.2. Mechanical Properties

**Figure 8:**
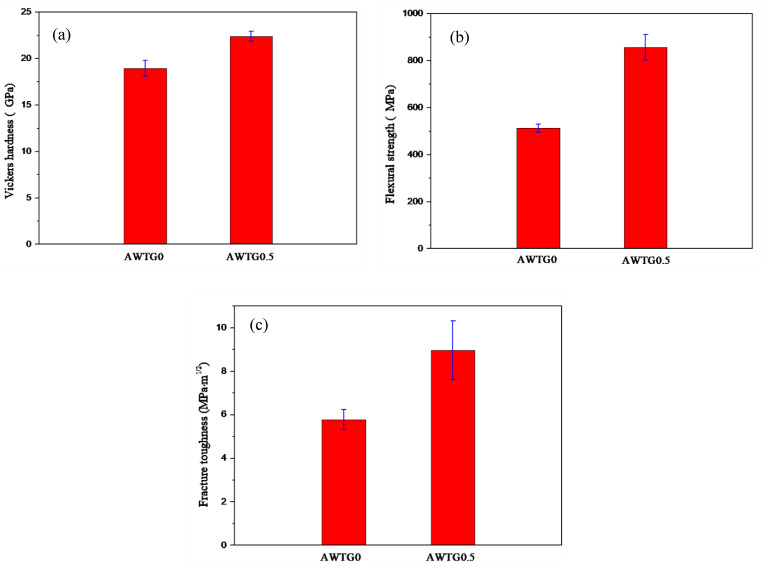
Mechanical properties of AWTG0 and AWTG0.5: (**a**) Vickers hardness, (**b**) flexural strength and (**c**) fracture toughness.

The mechanical properties of AWTG0 and AWTG0.5 are shown in Figure 8. It can be seen that the Vickers hardness, flexural strength and fracture toughness of AWTG0.5 are obviously higher than those of AWTG0. The improvement of Vickers hardness in AWTG0.5 can be attributed to the refined ceramic grains induced by the homogeneous distribution of graphene within the matrix [41]. The improvements of flexural strength and toughness in AWTG0.5 stem mainly from the formation of strong and weak hybrid interfaces. On the one hand, the strong interface can strengthen load transfer across the interface, thus improving strength, and on the other hand, the weak interface can induce more graphene pull-out, thus improving fracture toughness [6,9]. The detailed strengthening and toughening mechanisms will be discussed in the following sections. In addition, comparing the mechanical properties in this work and in reference [32], it can be concluded that the two-step HP sintering technology is beneficial to improving mechanical properties of Al_2_O_3_–WC–TiC–graphene ceramic composite comparing the traditional HP sintering technology.

### 3.3. Strengthening and Toughening Mechanisms

The strengthening efficiency (*R*) of graphene in composites can be calculated by the following equation [42]:

(4) $$R=\frac{\sigma_{c}-\sigma_{m}}{\sigma_{m}\cdot V_{\mathrm{graphene}}}$$

where *σ*_c_ is the strength of the composite, *σ*_m_ is the strength of the matrix, *V*_graphene_ is the volume fraction of graphene. According to Equation (4), the *R* value of AWTG0.5 can be determined as 135.18. This *R* value is relatively higher compared to the previous reports in graphene reinforced Al_2_O_3_-based ceramic composites [43,44,45], which indicates that graphene plays a good strengthening effect in this work.

**Figure 9:**
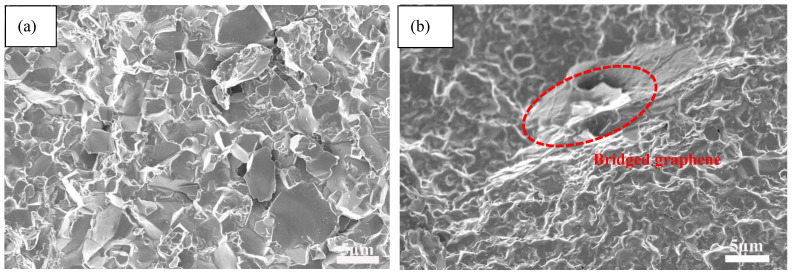
SEM images of fractured surfaces of (**a**) AWTG0 and (**b**) AWTG0.5. The red box circle indicates the bridged graphene.

Generally, the load transfer strengthening is considered to be a main strengthening mechanism in graphene reinforced composites [46,47,48]. As shown in Figure 9a, the fractured surface of AWTG0 is flatter overall, however, as shown in Figure 9b, the fractured surface of AWTG0.5 is characterized by the obvious dimples. Graphene provides a bridging function to connect the concave-convex parts, which effectively improves the load transfer capability of AWTG0.5 [49]. In addition, according to the shear-lag model, the high interfacial shear strength is beneficial to improving load transfer efficiency [50]. In Section 3.1, we proved that the graphene–WC and graphene–Al_2_O_3_ interfaces possess high interfacial shear strength, which further improves the load transfer capability of AWTG0.5.

According to the Hall–Petch relation, grain refinement can effectively improve the strength of composites. The improved strength contributed by the grain refinement can be calculated by the following equation [51]:

(5) $$\Delta \sigma_{\mathrm{Gr}}=K(d_{c}^{-0.5}-d_{m}^{-0.5})$$

where K is a constant, *d*_c_ and *d*_m_ are the average grain sizes of the composite with graphene and the composite without graphene, respectively. As shown in Table 2, the average grain size of AWTG0.5 (0.81 ± 0.45 μm) is far smaller than that of AWTG0 (1.56 ± 0.88 μm), therefore, the grain refinement is also a significant strengthening mechanism.

**Figure 10:**
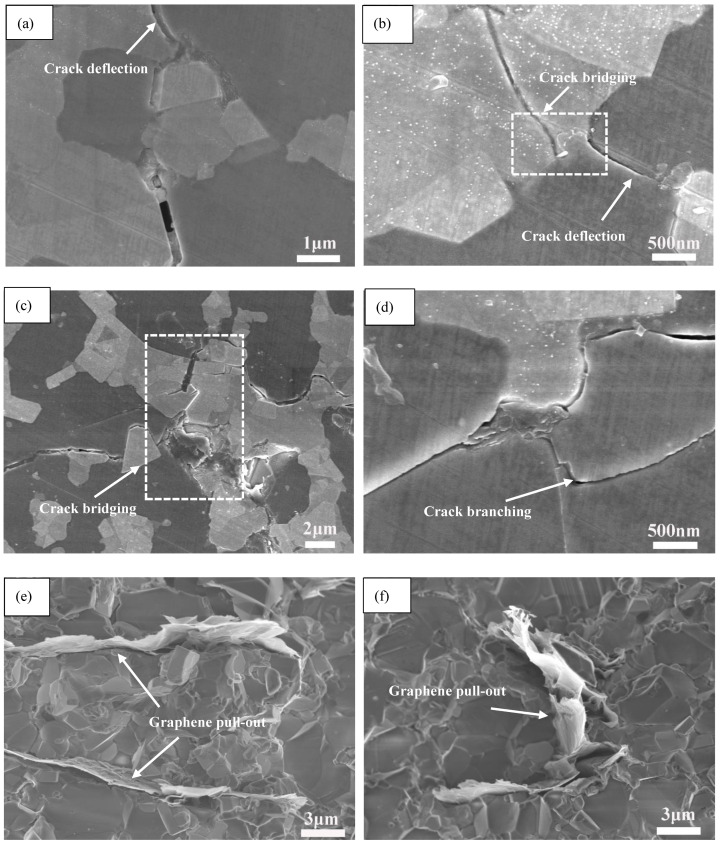
Toughening mechanisms induced by graphene in AWTG0.5. (**a**) Crack deflection, (**b**) crack deflection and crack bridging, (**c**) crack bridging, (**d**) crack branching, (**e**,**f**) graphene pull-out.

**Figure 11:**
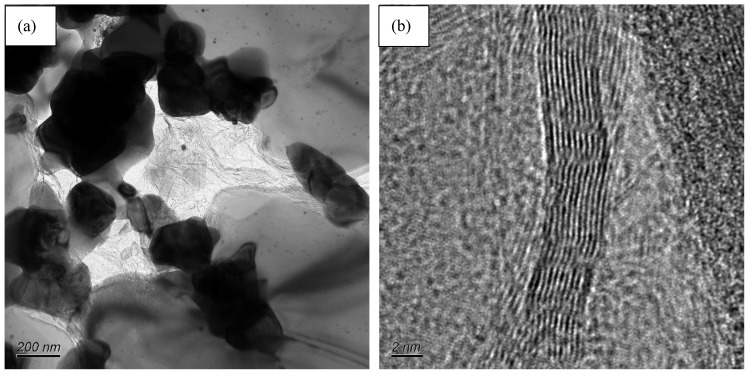
(**a**) TEM image of AWTG0.5, (**b**) HRTEM image of graphene in AWTG0.5. The wrinkled graphene can be observed in the two figures.

Figure 10 summarizes the main toughening mechanisms induced by graphene in AWTG0.5. As shown in Figure 10a,b, when cracks propagate to the regions with graphene, they are retarded by graphene. The cracks are hard to propagate along the previous direction and they are deflected to the regions without graphene along weak bonding interfaces. The crack deflection can dissipate fracture energy and improve fracture toughness. During the fracture of the ceramic composite with graphene, graphene is easy to detach from the matrix. When the interfacial bonding strength between graphene and ceramic grains is strong, graphene is difficult to pull out from the matrix, leading to the crack bridgings shown in Figure 10b,c, which can dissipate the fracture energy and prevent crack propagation [52]. However, when the bonding interface between graphene and ceramic grains is weak, as shown in Figure 10e,f, graphene will pull out from the matrix. The dissipated fracture energy of graphene pull-out can be evaluated by the following equation [53]:

(6) $$\Delta K_{IC}={\left(\frac{E\cdot A\cdot \tau_{i}}{r}\right)}^{\frac{1}{2}}\cdot l$$

where *E* is the Young’s modulus of the composite, *r* is the radius of graphene, *l* is the length of pulled out graphene, *τ_i_* is the friction of debonding interface, *A* is the area fraction of pulled out graphene. As a 2D nanomaterial, graphene possesses high specific surface area, the *A* value is much higher than other reinforcements. In addition, as shown in Figure 11, the graphene used in this work possesses obvious wrinkles, which can increase the friction of debonding interface. Therefore, graphene pull-out can dissipate more energy than other reinforced materials. Certainly, as shown in Figure 10d, the crack branching increases the crack propagation routes and dissipates fracture energy.

**Figure 12:**
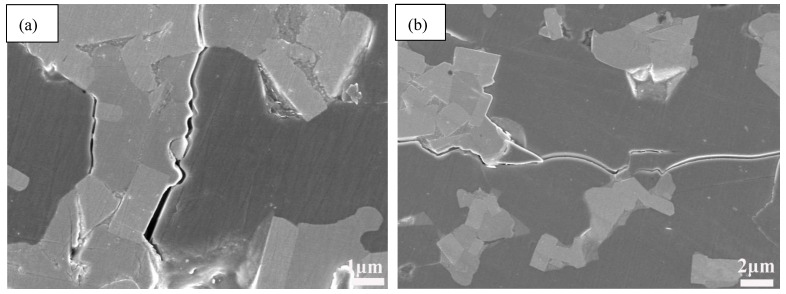
(**a**,**b**) SEM morphologies of Vickers indentation crack propagation in AWTG0.

Figure 12 shows the crack propagation morphologies of AWTG0, it can be seen that the cracks are relatively straight and they propagate along the boundaries between different grains. Therefore, it can be concluded that with the weak bonding interfaces it is hard to induce effective toughening mechanisms, however, the strong and weak hybrid interfaces induced by graphene can induce multiple toughening mechanisms.

## 4. Conclusions

The staggered distribution of strong and weak hybrid interfaces in ceramic composites is beneficial to improving the strength and toughness of ceramic materials. When graphene is incorporated into ceramic composites, depending on its unique 2D structure and large specific surface area, it can contact with different ceramic grains to create a lot of interfaces and facilitate the formation of strong-weak hybrid interfaces. Based on this material design philosophy, the Al_2_O_3_–WC–TiC–graphene composite was prepared by two-step hot pressing. The interfacial characteristics, mechanical properties as well as strengthening and toughening mechanisms were systematically investigated. The relationships among them were also analyzed.

Three different bonding interfaces including graphene–Al_2_O_3_, graphene–Al_2_OC and graphene–WC could be found in the Al_2_O_3_–WC–TiC–graphene composite by TEM. First-principles calculations were performed to investigate interfacial bonding strength. It was found that the adhesion energy and interfacial shear strength of the graphene–Al_2_OC interface were 0.287 eV/nm^2^ and 59.32 MPa, respectively, indicating the bonding interface was weak. However, the high adhesion and interfacial shear strength of the graphene–Al_2_O_3_ (0.967 eV/nm^2^, 395.77 MPa) and graphene–WC (0.781 eV/nm^2^, 229.84 MPa) interfaces indicated that the strong bonding interface was formed in the ceramic composite. Thus, the composite with the strong and weak hybrid interfaces was successfully fabricated. The microstructure analysis further demonstrated the formation of the strong and weak hybrid interfaces.

The strong and weak hybrid interfaces could induce strengthening mechanisms such as load transfer, grain refinement, etc., and toughening mechanisms such as crack bridging and graphene pull-out, etc. Therefore, mechanical properties of the Al_2_O_3_–WC–TiC–graphene composite were significantly improved compared to the Al_2_O_3_–WC–TiC composite.

This work can provide a method basis for the research on the interfacial structures and interfacial bonding strength of other ceramic matrix composites. Comparing the research results in this work and reference [32], it can be confirmed that the interfacial chemistry reaction products are subject to the dwell temperature, therefore, it is possible to realize interfacial regulation in ceramic matrix composites by adjusting dwell temperature. In addition, some conclusions about interfacial chemistry reaction products in this work, we think, are useful for the interfacial regulation investigations in other Al_2_O_3_-based ceramic composites.

## Figures and Tables

**Table 1:** Compositions of AWTG0 and AWTG0.5 (vol%).

Specimens	Al_2_O_3_	WC	TiC	MgO	Y_2_O_3_	Co	Graphene
AWTG0	72	18	6	0.5	0.5	3	0
AWTG0.5	71.5	18	6	0.5	0.5	3	0.5

**Table 2:** Relative density and average grain size of ceramic composites.

Specimens	Bulk Density (g/cm^3^)	Relative Density (%)	Average Grain Size (μm)
AWTG0	6.17 ± 0.07	98.41	1.56 ± 0.88
AWTG0.5	6.23 ± 0.02	99.52	0.81 ± 0.45

**Table 3:** The adhesion energy and interfacial shear strength of graphene–Al_2_OC, graphene–WC and graphene–Al_2_O_3_ interfaces.

Interfaces	Adhesion Energy (eV/nm^2^)	Interfacial Shear Strength (MPa)
Graphene–Al_2_OC	0.287	59.32
Graphene–WC	0.781	229.84
Graphene–Al_2_O_3_	0.967	395.77
